# Development and validation of a predictive nomogram for myopenia in patients with decompensated cirrhosis

**DOI:** 10.3389/fmed.2026.1841747

**Published:** 2026-08-19

**Authors:** Xiaona Yuan, Xin Yang, Xueming Du, Yangyang Zhou

**Affiliations:** 1 Department of Emergency Medicine, The First Affiliated Hospital of Zhengzhou University, Zhengzhou, China; 2 Department of Gynaecology and Obstetrics, The Third Affiliated Hospital of Zhengzhou University, Zhengzhou, China; 3 Department of Gastroenterology, Henan Provincial People’s Hospital, Zhengzhou, China; 4 Department of Gastroenterology, Union Hospital, Tongji Medical College, Huazhong University of Science and Technology, Wuhan, China

**Keywords:** corrected BMI, decompensated cirrhosis, myopenia, nomogram, prediction

## Abstract

**Background and aims:**

Myopenia is common in patients with cirrhosis and is associated with adverse clinical outcomes. However, current methods for assessing and diagnosing muscle depletion limit their application. In this study, we aimed to develop a simple and reliable nomogram to identify concurrent myopenia in patients with decompensated cirrhosis (DC).

**Methods:**

A total of 462 patients with DC at Wuhan Union Hospital between January 2015 and August 2022 were included. They were randomly divided into either the development cohort (*n* = 323) or the validation cohort (*n* = 139). Independent risk factors were screened to establish the risk prediction nomogram of myopenia using multivariate logistic regression analysis. Discrimination, accuracy, and clinical utility values were evaluated by receiver operating characteristic curves, calibration curves, decision curve analysis, and clinical impact plots.

**Results:**

Hematemesis, corrected body mass index (BMI), and fibrinogen were effectively identified as predictors of myopenia in patients with DC. The nomogram showed an area under the curve of 0.748 and 0.795 in the development and validation cohorts. The sensitivity, specificity, and Youden index of the development cohort were 47.7 and 89.2%, and 0.369, respectively. Calibration curves and decision curve analysis confirmed great accuracy and clinical utility.

**Conclusion:**

We developed and internally validated a nomogram to identify myopenia in patients with DC, which may support timely nutritional and functional evaluation.

## Introduction

Myopenia is common in patients with cirrhosis and is associated with adverse clinical outcomes. It has been reported that 37.5% of all patients with cirrhosis will develop myopenia, which was higher in male patients (1).

Regrettably, this critical condition is frequently overlooked, leading to treatment delays and potentially compromising patient outcomes. Earlier studies have revealed that patients with cirrhosis and myopenia had an increased risk of mortality and affected the surgical and post-transplantation outcomes (2–5). Furthermore, the major life-threatening complications of decompensated cirrhosis (DC), including ascites, spontaneous bacterial peritonitis, hepatic encephalopathy, and hepatorenal syndrome, have been shown to be influenced by myopenia. One recent meta-analysis has also corroborated these conclusions (1). Early identification and timely intervention could reduce the occurrence and development of myopenia (6). Thus, early identification of myopenia in DC patients is vital.

The gold standard for diagnosing myopenia is measurement of skeletal muscle mass area at the level of the third lumbar (L3) vertebra by computed tomography (CT) scan. However, it is not widely used in clinical practice because the routine use of CT scans, particularly if repeated over time, is limited due to the cost, radiation exposure, and time-consuming. Moreover, the diagnosis of myopenia requires the assistance of specially trained radiographers. Thus, new tools for screening and monitoring of such a condition would be required. Although some promising approaches were applied to assess skeletal muscle mass, including subjective global assessment (SGA), bioelectrical impedance analysis (BIA), or dual-energy X-ray absorptiometry (DXA), there are several drawbacks limiting their wide application (7, 8). Thus, a rigorous and easily available method for predicting and monitoring myopenia would be required.

Accordingly, we aimed to develop and internally validate a clinical nomogram to estimate the probability of myopenia in patients with decompensated cirrhosis and to identify individuals who may require formal skeletal muscle assessment. We hoped to use the nomogram model to identify myopenia populations so that we could provide timely intervention to reverse myopenia, eventually reducing socio-economic pressure and improving the quality of life of DC.

## Methods

### Patients

From January 2015 to August 2022, patients with DC at the Union Hospital of Tongji Medical College of Huazhong University of Science and Technology were enrolled, and their medical records were retrospectively collected and analyzed. This study has been approved by the Institutional Review Board of Wuhan Union Hospital, in accordance with the Declaration of Helsinki. Written informed consent was obtained from all patients. Inclusion criteria were as follows: (1) age between 18 and 85 and (2) clinically evident or pathology-proven DC. Exclusion criteria include (1) accompanied by severe heart-pulmonary, liver, or other organ dysfunction; (2) liver cancer or other malignant disease; (3) history of receiving a transjugular intrahepatic portosystemic shunt (TIPS), liver transplant, or splenic embolization; (4) without a CT scan within 3 months; and (5) insufficient clinical data. Finally, 462 patients were enrolled in our study. They were randomly allocated to either the development cohort (*n* = 323) or the validation cohort (*n* = 139) in a 7:3 ratio.

### Clinical definitions

CT image analysis at the third lumbar vertebra (L3) was applied to assess the skeletal muscle. Skeletal muscle index (SMI) was calculated by normalizing L3 cross-sectional muscle area to height in square meters (cm^2^/m^2^). Myopenia was diagnosed as SMI < 42 cm^2^/m^2^ for men and <38 cm^2^/m^2^ for women, which accords with the Japan Society of Hepatology guidelines for myopenia in liver disease (9). Cirrhosis was diagnosed histologically when liver biopsy results were available or clinically on the basis of concordant findings from the medical history, laboratory tests, endoscopy, and abdominal imaging. Clinical diagnosis required documented chronic liver disease together with evidence compatible with cirrhosis or portal hypertension, including: nodular liver morphology, splenomegaly, portosystemic collateral vessels, gastroesophageal varices, thrombocytopenia, or impaired hepatic synthetic function. DC was defined as cirrhosis with a current or documented previous episode of ascites, variceal bleeding, and/or overt hepatic encephalopathy. Each diagnosis was independently verified against the corresponding medical, endoscopic, and imaging records (10).

The index date was defined as the date of the CT examination used to assess the skeletal muscle index. Hematemesis was defined as documented vomiting of fresh blood or coffee-ground material occurring within 7 days before the index CT. Fibrinogen was defined as the laboratory value obtained closest to the index CT examination within 48 h. The neutrophil-to-lymphocyte ratio (NLR) was defined as the absolute neutrophil count divided by the absolute lymphocyte count. To minimize bias from fluid overload, a corrected dry weight was calculated by subtracting a fixed percentage of body weight based on the severity of ascites: 5% for mild, 10% for moderate, and 15% for severe ascites, plus an additional 5% if peripheral edema coexisted, in alignment with EASL clinical practice guidelines (11). Corrected BMI was subsequently computed as corrected dry weight (kg)/height^2^ (m^2^). The Model for End-Stage Liver Disease 3.0 (MELD 3.0) score and the Child–Pugh score and classification were determined as previously described (12).

### Data collection

The index date was defined as the date of the abdominal computed tomography examination used to assess skeletal muscle. Data available at or before the index date were retrospectively extracted from the electronic medical records using a standardized data-collection form.

Demographic and clinical variables included age, sex, ascites, history or occurrence of hematemesis, height, estimated dry body weight, and corrected body mass index. Laboratory variables included red blood cell count, hemoglobin, white blood cell count, neutrophil count, lymphocyte count, neutrophil-to-lymphocyte ratio, platelet count, platelet-to-lymphocyte ratio, alanine aminotransferase, aspartate aminotransferase, total bilirubin, total protein, albumin, creatinine, urea nitrogen, uric acid, prothrombin time, activated partial thromboplastin time, and fibrinogen. Liver disease severity was assessed using the Child–Pugh score and classification, and MELD 3.0. Radiological variables included the cross-sectional skeletal muscle area and skeletal muscle index measured at the third lumbar vertebral level.

### Statistical analysis

Continuous variables were assessed for normality using the Shapiro–Wilk test and visual inspection of histograms and Q–Q plots. As the continuous variables included in the present analysis were non-normally distributed, they are presented as medians with interquartile ranges and were compared between groups using the Mann–Whitney *U*-test. Categorical variables are presented as numbers and percentages and were compared using Pearson’s chi-square test. Fisher’s exact test was used when the expected frequency in any cell was less than 5. All statistical tests were two-sided.

Binary logistic regression analysis was performed to identify factors associated with myopenia. Univariable logistic regression analyses were initially conducted in the development cohort. Age and Child–Pugh score were retained in the multivariable model *a priori* because of their clinical relevance. Other candidate variables with *p*-values <0.05 in the univariable analysis were entered into a backward likelihood-ratio multivariable logistic regression model. The probability thresholds for variable removal and re-entry were 0.05 each. Multicollinearity was assessed using variance inflation factors before model fitting. The final model was constructed exclusively in the development cohort, and its coefficients were used to generate the nomogram. The model was subsequently evaluated in the validation cohort without refitting. Odds ratios are reported with 95% confidence intervals. A two-sided *p*-value of <0.05 was considered statistically significant.

ALT, AST, albumin, serum creatinine, blood urea nitrogen, and activated partial thromboplastin time were categorized according to the hospital laboratory’s reference ranges. For corrected BMI, platelet count, and fibrinogen, receiver operating characteristic curve (ROC) analyses were performed exclusively in the development cohort. The optimal cutoff value for each variable was defined as the value that maximized the Youden index, calculated as the sum of sensitivity and specificity minus 1. The resulting thresholds were corrected BMI < 21.9 kg/m^2^, platelet count >54.5 × 10^9^/L, and fibrinogen >2.26 g/L. These thresholds were subsequently applied unchanged to the validation cohort. ROC was used to assess the nomogram’s discriminatory ability. The calibration curves and decision curve analysis (DCA) were plotted to assess the nomograms’ predictive ability and clinical value. Statistical analysis was conducted with SPSS 26.0 (IBM Corp., Armonk, New York, USA) and R software (version 4.2.1; The R Foundation, Vienna, Austria). A *p*-value of < 0.05 was considered statistically significant.

## Results

### Patient characteristics

**Figure 1:**
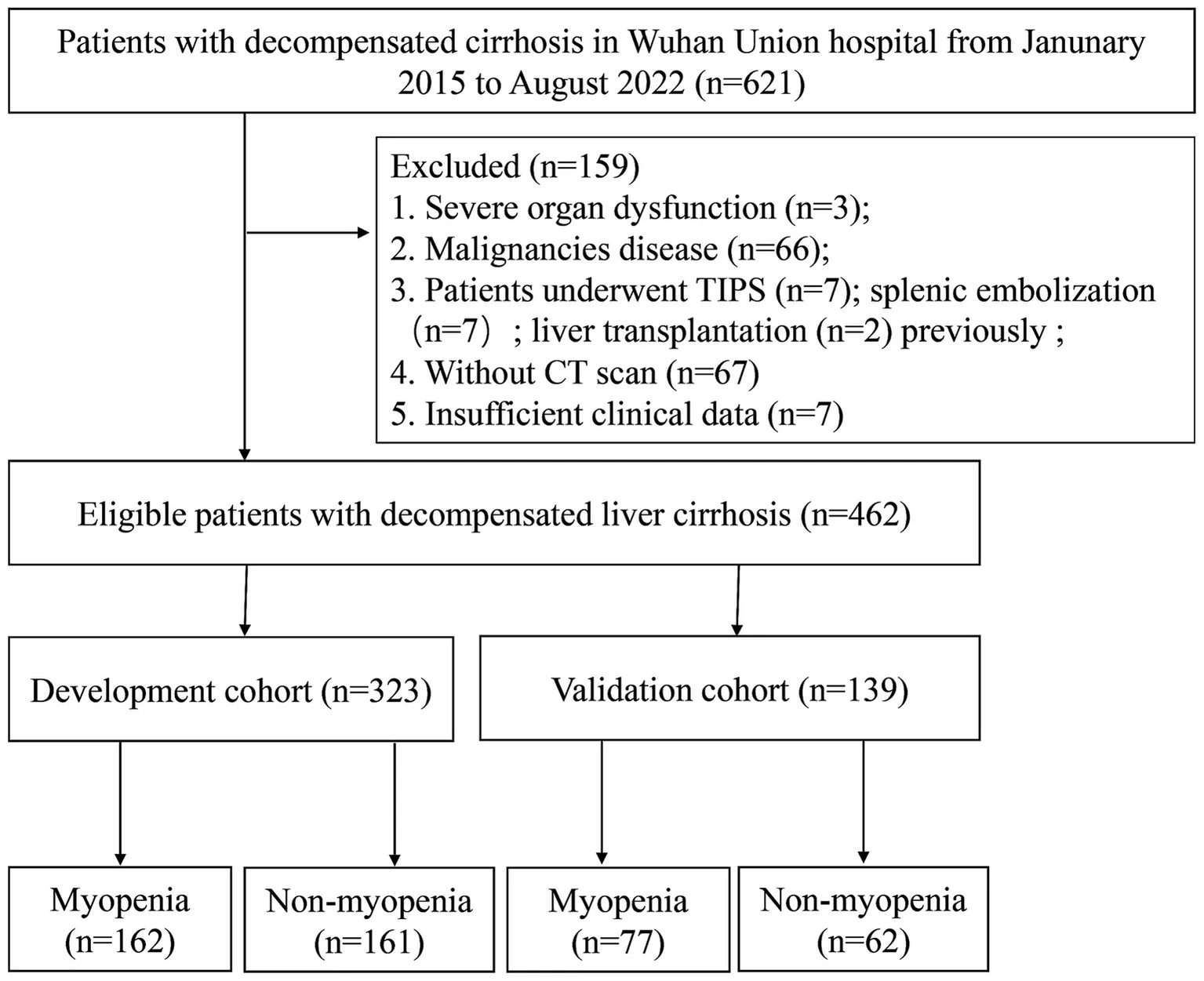
Flowchart of this study.

A total of 462 patients were eventually enrolled in this study and were randomly assigned to the development (*n* = 323) and the validation cohort (*n* = 139) in a 7:3 ratio, as shown in Figure 1. Baseline characteristics and laboratory tests of patients with DC are shown in Table 1 and Supplementary Table 1. In total, 281 (60.82%) were male, with a median age of 54 years. 69.05% of patients were classified as Child–Pugh B and C. No correlation was found between decreased skeletal muscle index and age (*r* = −0.05; *p* = 0.26) (Supplementary Figure 1), and there were no significant differences in the median age of the two analyzed groups (myopenia vs. non-myopenia group was 54 vs. 55 years; *p* = 0.369). The prevalence of myopenia was 51.73% (*n* = 239), with no difference between males and females.

**Table 1:** Associations between characteristics of myopenia and non-myopenia patients with decompensated cirrhosis in the development and validation cohort.

Variable	Development cohort			Validation cohort		
	Non-myopenia group (*n* = 161)	Myopenia group (*n* = 162)	*P*-value	Non-myopenia group (*n* = 62)	Myopenia group (*n* = 77)	*P*-value
Age, years	52.00 (46.00, 61.00)	55.00 (48.00, 63.50)	0.459^a^	54.50 (48.75, 63.00)	55.00 (47.00, 66.00)	0.718^a^
Gender			0.385^b^			0.218^b^
Male	102 (63.35%)	95 (58.64%)		41 (66.13%)	43 (55.84%)	
Female	59 (36.65%)	67 (41.36%)		21 (33.87%)	34 (44.16%)	
Ascites	102 (63.35%)	92 (56.79%)	0.228^b^	42 (67.74%)	46 (59.74%)	0.331^b^
Hematemesis	75 (46.58%)	97 (59.88%)	**0.017** ^b^	24 (38.71%)	54 (70.13%)	**<0.001** ^b^
Corrected BMI	22.96 (21.48, 24.34)	21.24 (19.59, 22.89)	**<0.001** ^a^	23.08 (22.07, 24.62)	21.48 (20.52, 23.04)	**<0.001** ^a^
Corrected BMI < 21.9	48 (29.81%)	103 (63.58%)	**<0.001** ^b^	14 (22.58%)	48 (62.34%)	**<0.001** ^b^
RBC	3.25 (2.66, 3.69)	3.15 (2.74, 3.79)	0.918^a^	3.32 (2.34, 3.86)	3.14 (2.68, 3.70)	0.759^a^
HGB	95.00 (74.00, 113.00)	92.00 (75.00, 115.00)	0.575^a^	91.50 (67.75, 119.50)	91.00 (75.00, 110.50)	0.887^a^
WBC	3.32 (2.36, 4.80)	3.59 (2.41, 5.59)	0.482^a^	3.46 (2.45, 4.59)	3.67 (2.76, 6.22)	0.123^a^
Neutrophil count	1.87 (1.32, 3.00)	1.95 (1.26, 3.17)	0.839^a^	2.03 (1.28, 3.06)	2.08 (1.53, 4.24)	0.131^a^
Lymphocyte	0.84 (0.58, 1.28)	1.00 (0.65, 1.44)	**0.034** ^a^	0.80 (0.57, 1.29)	1.09 (0.60, 1.44)	0.225^a^
NLR	2.33 (1.54, 3.40)	2.07 (1.45, 3.09)	0.149^a^	2.30 (1.70, 3.60)	2.55 (1.67, 4.16)	0.444^a^
PLT	77.00 (54.00, 128.00)	87.00 (65.00, 154.00)	**0.012** ^a^	67.50 (49.00, 117.50)	88.00 (56.50, 163.50)	**0.025** ^a^
PLT > 54.5	118 (73.29%)	141 (87.04%)	**0.002** ^b^	40 (64.52%)	62 (80.52%)	**0.034** ^b^
PLR	94.29 (66.22, 133.01)	103.33 (72.03, 140.83)	0.319^a^	85.60 (59.84, 121.62)	106.12 (66.94, 137.35)	0.080^a^
ALT	29.00 (20.00, 42.00)	26.00 (18.00, 38.00)	0.120^a^	29.00 (19.00, 36.75)	26.00 (18.00, 37.00)	0.369^a^
AST	37.00 (27.00, 49.00)	36 (25.00, 53.00)	0.545^a^	37.00 (27.00, 48.25)	33.00 (24.00, 42.50)	0.111^a^
TBIL	19.00 (13.10, 28.70)	18.80 (12.90, 25.85)	0.313^a^	20.70 (15.30, 27.93)	19.50 (14.05, 26.60)	0.556^a^
Total protein	61.60 (56.20, 66.40)	62.30 (57.00, 68.70)	0.165^a^	61.65 (56.90, 70.43)	61.60 (54.80, 68.05)	0.794^a^
ALB	33.00 (28.80, 37.10)	34.50 (30.65, 37.90)	0.062^a^	33.85 (28.88, 37.63)	33.50 (29.40, 37.40)	0.950^a^
Creatine	62.40 (51.10, 73.50)	63.60 (53.75, 74.10)	0.658^a^	63.75 (57.75, 76.63)	62.70 (53.10, 73.35)	0.460^a^
Urea nitrogen	4.99 (4.09, 6.57)	5.10 (4.00, 6.99)	0.794^a^	4.98 (3.91, 6.69)	4.89 (3.82, 6.68)	0.806^a^
Uric acid	269.70 (216.60, 324.60)	275.70 (216.80, 352.20)	0.733^a^	279.65 (221.83, 331.08)	274.00 (217.85, 353.25)	0.901^a^
APTT	39.20 (36.40, 41.90)	38.50 (36.20, 42.45)	0.691^a^	38.70 (35.55, 42.63)	37.70 (35.75, 41.65)	0.518^a^
Fibrinogen	2.00 (1.59, 2.43)	2.22 (1.81, 2.70)	**0.005** ^a^	2.00 (1.50, 2.59)	2.27 (1.72, 2.88)	0.067^a^
Fibrinogen >2.26	44 (27.33%)	88 (54.32%)	**<0.001** ^b^	21 (33.87%)	43 (55.84%)	**0.010** ^b^
Child–Pugh score	8.00 (7.00, 9.00)	7.00 (6.00, 8.00)	0.057a	8.00 (6.00, 8.75)	8.00 (6.00, 9.00)	0.594^a^
Child–Pugh class			**0.030** ^b^			0.552^b^
A	38 (23.60%)	58 (35.80%)		21 (33.87%)	26 (33.76%)	
B	97 (60.25%)	88 (54.32%)		33 (53.23%)	36 (46.75%)	
C	26 (16.15%)	16 (9.88%)		8 (12.90%)	15 (19.48%)	
MELD 3.0	19.00 (15.00, 26.00)	22.00 (17.00, 29.00)	**0.013** ^a^	18.00 (16.00, 25.00)	25.00 (17.00, 29.00)	0.160^a^

Continuous variables are presented as median (interquartile range) and were compared using the Mann–Whitney *U*-test. Categorical variables are presented as number (percentage) and were compared using Pearson’s chi-square test or Fisher’s exact test, as appropriate. Superscript “a” indicates the Mann–Whitney *U*-test, “b” indicates Pearson’s chi-square test, and “c” indicates Fisher’s exact test. All tests were two-sided. Bold values indicate *P* < 0.05. BMI: body mass index; RBC: red blood cell; HBG: hemoglobin; WBC: white blood cell; NLR: neutrophil-to-lymphocyte ratio; PLT: platelet; PLR: platelet-to-lymphocyte ratio; ALT: alanine aminotransferase; AST: aspartate aminotransferase; TBIL: total bilirubin; ALB: albumin; APTT: activated partial thromboplastin time; MELD 3.0: Model for end-stage liver disease version 3.

### Risk factors for myopenia

All the analyzed variables contained complete data. ROC analysis conducted in the development cohort identified 21.9 kg/m^2^ as the optimal cutoff for corrected BMI, 54.5 × 10^9^/L for platelet count, and 2.26 g/L for fibrinogen, based on the maximum Youden index. These thresholds were applied without modification in the validation cohort. Furthermore, the development cohort was used for all factor analyses and development of the nomogram. In univariate analysis, hematemesis, corrected BMI < 21.9, platelet >54.5, ALB < 35, and fibrinogen > 2.26 were significantly different between the two groups (*p* < 0.05), as shown in Table 2.

**Table 2:** Univariate and multivariate analyses of variables for myopenia in the development cohort.

Variable	Myopenia			
	Univariate analysis		Multivariate analysis	
	OR (95% CI)	*P*	OR (95% CI)	*P*
Age, years	1.007 (0.989, 1.024)	0.458	1.011 (0.992, 1.031)	0.259
Child–Pugh score	0.876 (0.761, 1.008)	0.065	1.051 (0.845, 1.306)	0.657
Gender (male)	1.219 (0.779, 1.908)	0.386		
Hematemesis	1.711 (1.101, 2.660)	**0.017**	1.796 (1.077, 2.995)	**0.025**
Ascites	0.760 (0.486, 1.188)	0.229		
Corrected BMI < 21.9	2.644 (1.687, 4.143)	**<0.001**	3.900 (2.361, 6.445)	**<0.001**
Platelet >54.5	2.447 (1.375, 4.354)	**0.002**	1.880 (0.978, 3.614)	0.058
ALT >2ULN	0.561 (0.215, 1.463)	0.237		
AST > 2ULN	0.763 (0.373, 1.559)	0.458		
ALB < 35 g/L	0.639 (0.409, 0.996)	**0.048**	0.650 (0.329, 1.283)	0.214
Creatinine > 1ULN	0.491 (0.089, 2.717)	0.415		
Urea nitrogen > 1 ULN	1.225 (0.728, 2.061)	0.445		
APTT > 1 ULN	0.878 (0.552, 1.395)	0.581		
Fibrinogen >2.26	3.162 (1.987, 5.031)	**<0.001**	2.975 (1.733, 5.107)	**<0.001**

Odds ratios and corresponding 95% confidence intervals were estimated using binary logistic regression analysis. Age and Child–Pugh score were retained in the multivariable model a priori as clinically relevant covariates. Other candidate variables with *P* < 0.05 in the univariable analysis were entered into a backward likelihood ratio multivariable logistic regression model. The removal and re-entry criteria were *p* > 0.05 and *P* < 0.05, respectively. OR: odds ratio; CI: confidence intervals; BMI: body mass index; ALT: alanine aminotransferase; AST: aspartate aminotransferase; ALB: albumin; APTT: activated partial thromboplastin time; ULN: upper limit of normal. Bold values indicate *p*< 0.05.

After mandatory adjustment for age and Child–Pugh score, hematemesis, corrected BMI, and fibrinogen remained independently associated with myopenia. The results revealed that hematemesis, corrected BMI < 21.9, and fibrinogen > 2.26 were independent risk factors for myopenia in patients with DC.

### Construction and validation of the predictive nomogram

**Figure 2:**
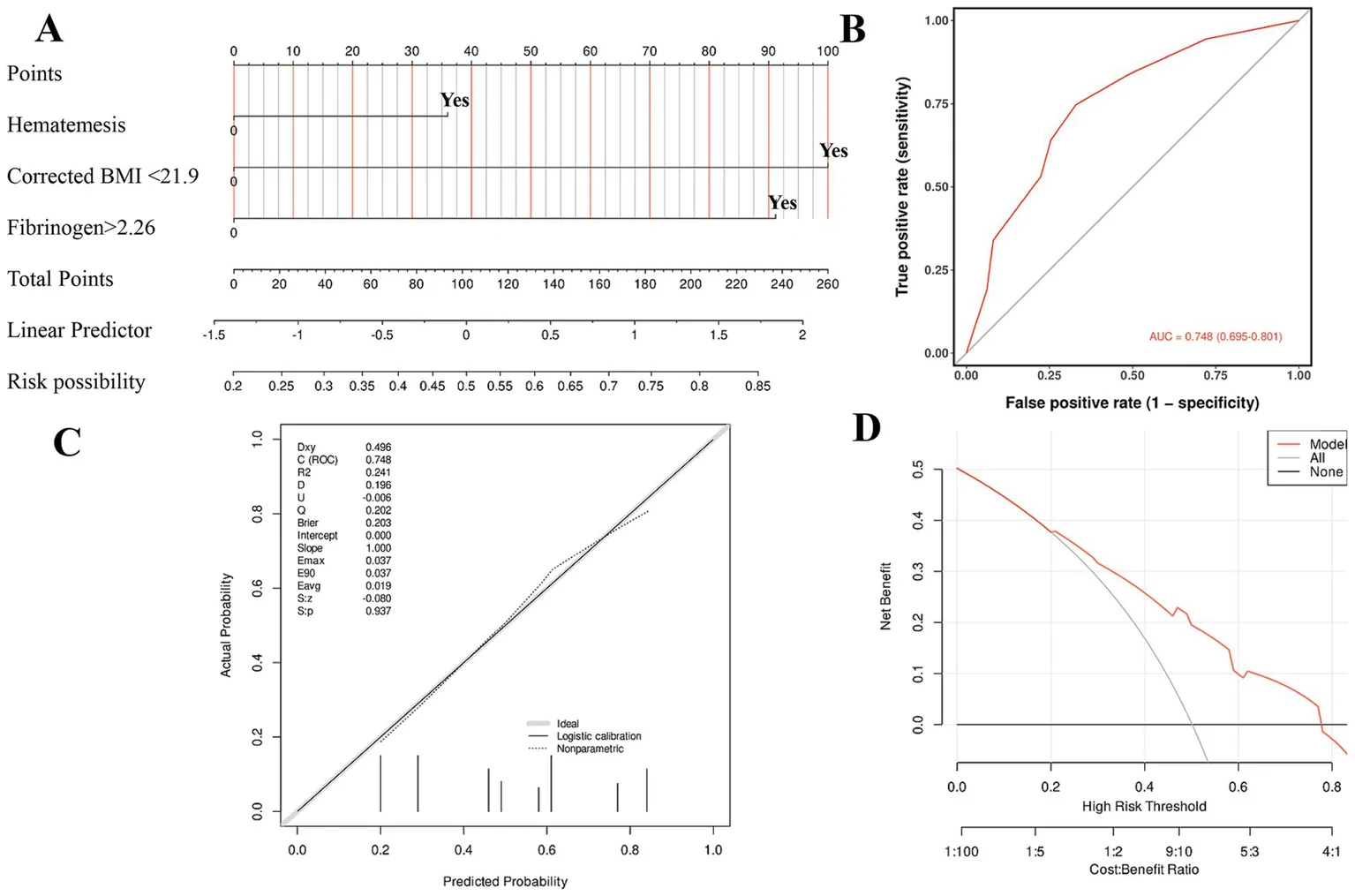
Development of a nomogram for predicting myopenia in patients with DC. **(A)** A nomogram for predicting myopenia risk. To use this diagnostic tool, locate the patient’s status for each of the three clinical variables along their respective axes: hematemesis, corrected BMI, and fibrinogen. Draw a straight vertical line upward from each variable’s value to the top “Points” axis to determine the score assigned to that factor. Sum the individual points from all three risk factors to calculate the “Total Points.” Finally, locate this sum on the “Total Points” axis and draw a vertical line straight down to the “Risk of Low Muscle Mass” axis to read the corresponding individualized probability. **(B)** The ROC of the nomogram in the development cohort. **(C)** Calibration curve of the development cohort. **(D)** Decision curve analysis for the development cohort. DC: Decompensated cirrhosis; AUC: Area under the curve; BMI: Body mass index; ROC: Receiver operating characteristic.

Based on the results of logistic regression analyses, hematemesis, corrected BMI < 21.9, and fibrinogen > 2.26 were used to construct the myopenia predictive nomogram as shown in Figure 2A. The corresponding score for each factor was obtained by crossing to the nomogram, and the total score was summed to predict the risk of developing myopenia in patients with DC.

The validation of this prediction model was performed by area under the curve (AUC) and the calibration curve. In the development cohort, the AUC of the nomogram was 0.748 (95% CI 0.695–0.801) (Figure 2B), with a sensitivity, specificity, and Youden index of 47.7 and 89.2%, and 0.369, respectively. The AUC of hematemesis, corrected BMI < 21.9, and fibrinogen > 2.26 were 0.566, 0.669, and 0.635, respectively. Similarly, the AUC of the validation cohort was 0.795 (95% CI 0.721–0.869) (Supplementary Figure 2A), with a sensitivity, specificity, and Youden index of 73.6 and 72.9%, and 0.465, respectively.

In the development and validation cohorts, the calibration curve showed agreement between the observation and prediction, as shown in Figure 2C and Supplementary Figure 2B. The results showed the nomogram was still valid for use in the validation cohorts, and the calibration curve of this model was relatively close to the ideal curve, indicating the predicted results were consistent with the actual findings.

### Clinical utility of the nomogram

The clinical effectiveness of this predictive model was assessed using DCA. The DCA for the development and validation cohorts is shown in Figure 2D and Supplementary Figure 2C, which revealed that patients could benefit from this novel predictive model when the threshold was set to 20 ~ 70% and 20 ~ 80% for the development and validation cohorts, respectively.

## Discussion

Myopenia is a progressive, generalized skeletal muscle disorder characterized by accelerated loss of muscle mass, strength, and physiological function. Recently, there has been increased attention to myopenia, which was a complication of cirrhosis. The incidence of myopenia in patients with cirrhosis was 40 to 70% (5, 13–15). The presence of myopenia has been recognized as an important concern in patients with cirrhosis due to the association with higher mortality, unfavorable surgical and post-transplantation outcomes, and increased risk of other complications (16).

While it is easy to visually identify myopenia in its advanced stages, objective and validated tools to detect muscle loss in its early stages are needed, as timely therapeutic interventions have the potential to modify and mitigate the progression of myopenia, thus improving the prognosis of these patients. In this regard, SMI-based CT scanning is a validated and recommended method in the diagnosis of myopenia. However, the utilization of CT scans as a regular practice, especially for repetitive assessments to monitor the effectiveness of treatments, is limited in clinical settings owing to a variety of factors, including cost, radiation exposure, and time-consuming. Given the clinical setting, a universal, convenient, fast, and less expensive model to diagnose myopenia is warranted.

In this study, we developed and validated a simple and convenient nomogram to predict the risk of myopenia in patients with DC. The AUC of the nomogram model was 0.748 and 0.795 in the development and validation datasets, respectively. Characteristics eventually included in this model were hematemesis, corrected BMI, and fibrinogen. These factors are economically accessible and easily available, which makes them suitable for myopenia screening in primary hospitals. In addition, the results of the calibration curve and DCA curve proved the reliability and accuracy of the model. Therefore, this model could realize early identification of patients with DC at risk of myopenia, and timely adopt interventions to prevent or slow its development and progression.

Recently, some studies on the risk factors of myopenia have been reported (17, 18). However, these studies were mainly based on European data. There are differences in body size, eating habits, and lifestyle among different regions, as well as in the threshold value used to define myopenia. Thus, the results from European countries may not be suitable for Asian countries. We developed this simple nomogram based on data from China to predict the risk of myopenia.

For the present study, hematemesis, corrected BMI, and fibrinogen were identified as independent prognostic factors. Hematemesis was regarded as a prognostic factor of myopenia, which was easy to understand. Hematemesis is one of the clinical manifestations of patients with DC and is also one of the main causes of death (17, 18). Meanwhile, myopenia is also a major factor in predicting prognosis. Therefore, hematemesis could indicate myopenia. The association between hematemesis and myopenia should be interpreted cautiously. Hematemesis may reflect clinically significant portal hypertension, acute blood loss, reduced oral intake, hospitalization, and catabolic stress; however, variation in the timing, etiology, and treatment of bleeding may have resulted in residual confounding. Hematemesis should therefore be regarded as an associated clinical marker rather than a direct cause of myopenia.

BMI, an easy and immediate index of health, was commonly applied in clinical practice. Several studies have proved that low BMI increased the risk of worse outcomes (19, 20). Our study also found that corrected BMI < 21.9 had a higher risk of myopenia, which was consistent with previous studies (21). The association between lower corrected BMI and myopenia is biologically plausible. A lower BMI may reflect diminished energy stores, inadequate protein and caloric intake, and advanced nutritional depletion. Cirrhosis is characterized by reduced hepatic glycogen storage and an accelerated starvation response, resulting in earlier reliance on lipid oxidation and amino acid-derived gluconeogenesis during fasting. Increased amino acid utilization may contribute to a persistent negative muscle protein balance. However, corrected BMI remains an indirect and imperfect marker of body composition. In particular, ascites and peripheral edema may artificially increase body weight, whereas patients with obesity may have substantial muscle depletion despite a normal or elevated BMI. Accordingly, the present model uses fluid-adjusted BMI as an accessible screening variable.

Fibrinogen is an acute-phase protein that is produced by hepatocytes, indicating a proinflammatory state (22). The positive association between fibrinogen and low skeletal muscle mass may indicate that systemic inflammation contributes to muscle depletion. Proinflammatory signaling may impair anabolic pathways and enhance proteolytic processes, thereby reducing muscle protein synthesis and increasing muscle breakdown. Nevertheless, interpretation of fibrinogen in DC is complex. Advanced hepatic dysfunction may reduce fibrinogen synthesis, whereas infection, tissue injury, acute bleeding, and other inflammatory stimuli may increase its concentration. Measurements may also be affected by transfusion or treatment administered around the time of an acute bleeding episode. Thus, elevated fibrinogen may represent a context-dependent marker of an inflammatory or acute decompensating phenotype rather than a direct mediator of muscle loss. Prospective studies with serial measurements of fibrinogen and other inflammatory biomarkers are needed to clarify this relationship.

Aging is a well-recognized contributor to the decline in skeletal muscle mass and function (23). Nevertheless, age was not significantly associated with myopenia in the present cohort. This finding should not be interpreted as indicating that age is biologically irrelevant. One possible explanation is that our study population was predominantly middle-aged, with a median age of 54 years, thereby providing limited separation between younger and older patients. Moreover, all participants had decompensated cirrhosis, in whom muscle depletion may be driven predominantly by disease-specific mechanisms, including inadequate dietary intake, accelerated starvation, systemic inflammation, endocrine abnormalities, and recurrent decompensating events. These mechanisms may induce substantial secondary muscle loss even in relatively young or middle-aged patients, thereby attenuating the apparent effect of chronological age. In addition, the cross-sectional design captured muscle mass at one time point and could not evaluate longitudinal age-related changes. Further prospective studies incorporating a broader age distribution, serial muscle measurements, and functional assessments are required to clarify the age–muscle relationship in patients with DC.

Several models have previously been proposed to identify low skeletal muscle mass in patients with cirrhosis. Tandon et al. developed sex-specific nomograms incorporating BMI and ultrasound-measured thigh muscle thickness (24). The Sarcopenia HIBA score was subsequently developed in patients on the liver transplant waiting list and incorporated male sex, BMI, Child–Pugh score, and the serum creatinine-to-cystatin C ratio (25). The latter model showed an AUC of 0.862 after internal validation by bootstrapping. These models support the feasibility of identifying patients at increased risk of low muscle mass without repeated CT-based body composition measurements. However, they require measurements that are not routinely available in all clinical settings and were developed in populations that differ from the present cohort.

The generalizability of the present nomogram requires careful consideration. The model was developed and internally evaluated in a single-center cohort of Chinese patients with DC. The nomogram may have screening value in populations clinically similar to the present cohort, but its performance and absolute probability estimates cannot be assumed to remain unchanged in other settings.

Transportability may be affected by differences in ethnicity, body composition, nutritional status, etiology and severity of cirrhosis, prevalence of low skeletal muscle mass, management practices, and laboratory measurement procedures. In addition, CT-defined low skeletal muscle mass was determined using sex-specific skeletal muscle index thresholds proposed for patients with liver disease in an Asian population. Alternative thresholds or muscle-assessment criteria used in other regions may result in different outcome classifications and baseline prevalence. Such differences could affect both model calibration and the relative contribution of individual predictors.

Several limitations should be acknowledged in this study. First, this retrospective study was performed in a single center, which might lead to bias; large multicenter prospective studies are needed in the future. Second, the developed nomogram model was only validated internally, without external validation. Third, this study excluded several cases without CT or clinical data, potentially introducing selection bias. Fourth, muscle strength and physical performance were not routinely assessed because of the retrospective design. Therefore, the present study evaluated CT-defined myopenia rather than consensus-defined sarcopenia. Although L3 skeletal muscle index provides an objective measure of muscle quantity, it does not capture muscle strength or functional impairment. Future prospective studies should incorporate handgrip strength and standardized physical-performance assessments to determine whether the model can identify patients with confirmed sarcopenia.

## Conclusion

In this study, we developed a simple model for predicting myopenia that included hematemesis, corrected BMI, and fibrinogen, all of which were readily obtained. Importantly, the prediction model showed simplicity, convenience, and timely applicability. Therefore, this simple and efficient model might be more favorable for use in clinical practice and could achieve early detection of myopenia.

## Data Availability

The raw data supporting the conclusions of this article will be made available by the authors, without undue reservation.
