# Supplementary material for: Development and validation of a predictive nomogram for myopenia in patients with decompensated cirrhosis
Source: Front Med (Lausanne). 2026 Aug 19;13:1841747. doi: 10.3389/fmed.2026.1841747 (PMC13535287; doi:10.3389/fmed.2026.1841747)
Supplement: Supplementary file 1 [file Data_Sheet_1.DOC]

**Supplementary Table 1** Baseline characteristics of the development and validation cohorts

| Variables | Total  (n=462) | Development Cohort  (n=323) | Validation Cohort  (n=139) | *P* value |
| --- | --- | --- | --- | --- |
| Age, years | 54.00 (47.00, 63.00) | 54.00 (47.00, 62.00) | 55.00 (48.00, 63.00) | 0.430^a^ |
| Gender |  |  |  | 0.910^b^ |
| Male | 281 (60.82%) | 197 (60.99%) | 84 (60.43%) |  |
| Female | 181 (39.18%) | 126 (39.01%) | 55 (39.57%) |  |
| Ascites | 282 (61.04%) | 194 (60.06%) | 88 (63.31%) | 0.512^b^ |
| Hematemesis | 250 (54.11%) | 172 (53.25%) | 78 (56.12%) | 0.571^b^ |
| Corrected BMI | 22.21 (20.60, 24.01) | 22.04 (20.50, 23.88) | 22.59 (20.90, 24.02) | 0.162^a^ |
| Myopenia | 239 (51.73%) | 162 (50.15%) | 77 (55.40%) | 0.301^b^ |
| RBC | 3.21 (2.69, 3.78) | 3.20 (2.73, 3.77) | 3.21 (2.57, 3.78) | 0.740^a^ |
| HGB | 93.00 (74.00, 115.00) | 93.00 (75.00, 115.00) | 91.00 (72.00, 115.00) | 0.572^a^ |
| WBC | 3.43 (2.45, 5.31) | 3.38 (2.38, 5.19) | 3.62 (2.59, 5.38) | 0.374^a^ |
| Neutrophilic | 1.96 (1.38, 3.20) | 1.92 (1.28, 3.16) | 2.07 (1.45, 3.47) | 0.252^a^ |
| Lymphocyte | 0.92 (0.60, 1.36) | 0.91 (0.62, 1.36) | 0.94 (0.60, 1.36) | 0.791^a^ |
| NLR | 2.25 (1.50, 3.47) | 2.19 (1.50, 3.27) | 2.43 (1.68, 3.85) | 0.069^a^ |
| PLT | 82.00 (57.00, 139.25) | 82.00 (60.00, 139.00) | 80.00 (53.00, 138.00) | 0.312^a^ |
| PLR | 98.79 (67.50, 133.77) | 100.00 (68.48, 135.45) | 95.24 (64.71, 130.61) | 0.485^a^ |
| ALT | 27.00 (19.00, 40.00) | 28.00 (19.00, 41.00) | 27.00 (19.00, 37.00) | 0.347^a^ |
| AST | 35.00 (26.00, 49.00) | 36.00 (26.00, 51.00) | 34.00 (26.00, 46.00) | 0.332^a^ |
| TBIL | 19.20 (13.38, 26.88) | 18.80 (12.90, 26.80) | 20.20 (14.50, 27.10) | 0.105^a^ |
| Total protein | 61.90 (56.58, 67.60) | 62.20 (56.70, 67.40) | 61.60 (55.90, 68.40) | 0.775^a^ |
| ALB | 33.75 (29.40, 37.43) | 33.80 (29.50, 37.50) | 33.60 (29.40, 37.40) | 0.877^a^ |
| Creatine | 63.35 (53.20, 73.93) | 63.40 (52.40, 74.00) | 63.30 (55.10, 73.90) | 0.709^a^ |
| Urea nitrogen | 5.00 (4.00, 6.76) | 5.06 (4.05, 6.78) | 4.92 (3.89, 6.70) | 0.540^a^ |
| Uric acid | 273.90 (216.90, 341.30) | 271.40 (216.80, 341.50) | 276.80 (219.60, 340.70) | 0.780^a^ |
| PT | 15.50 (14.30, 16.80) | 15.50 (14.30, 16.80) | 15.70 (14.45, 16.85) | 0.609^a^ |
| APTT | 38.70 (36.18, 42.13) | 39.10 (36.30, 42.20) | 38.30 (35.80, 41.90) | 0.359^a^ |
| Fibrinogen | 2.14 (1.65, 2.63) | 2.13 (1.65, 2.59) | 2.17 (1.61, 2.68) | 0.776^a^ |
| Child Pugh score | 7.00 (6.00, 9.00) | 7.00 (6.00, 8.00) | 8.00 (6.00, 9.00) | 0.582^a^ |
| Child Pugh class |  |  |  | 0.298^b^ |
| A | 143 (30.95%) | 96 (29.72%) | 47 (33.81%) |  |
| B | 254 (54.98%) | 185 (57.28%) | 69 (49.64%) |  |
| C | 65 (14.07%) | 42 (13.00%) | 23 (16.55%) |  |
| MELD | 21.00(16.00, 28.00) | 21.00 (15.00, 29.00) | 21.00 (16.00, 27.00) | 0.827^a^ |

Note: Continuous variables are presented as median (interquartile range) and were compared using the Mann–Whitney U test. Categorical variables are presented as number (percentage) and were compared using Pearson’s chi-square test or Fisher’s exact test, as appropriate. Superscript “a” indicates the Mann–Whitney U test, “b” indicates Pearson’s chi-square test, and “c” indicates Fisher’s exact test. All tests were two-sided. Bold values indicate *P* <0.05. BMI: body mass index; RBC: Red blood cell; HBG: Hemoglobin; WBC: White blood cell; NLR: Neutrophilic lymphocyte ratio; PLT: Platelet; PLR: platelet-to-lymphocyte ratio; ALT: Alanine aminotransferase; AST: Aspartate aminotransferase; TBIL: Total bilirubin; ALB: Albumin; APTT: activated partial thromboplastin time; INR: International normalized ratio; MELD 3.0: Model for end stage liver disease version 3.

**Supplementary Table 2** Univariate and multivariate analysis of variables for myopenia in validation cohort

| Variable | Myopenia | | | |
| --- | --- | --- | --- | --- |
|  | Univariate analysis | | Multivariate analysis | |
|  | OR (95% CI) | *p* | OR (95% CI) | *p* |
| Age, years | 1.005 (0.978, 1.033) | 0.724 | 0.996 (0.962, 1.031) | 0.815 |
| Child Pugh score | 1.076 (0.890, 1.301) | 0.449 | 1.244 (0.876, 1.765) | 0.222 |
| Gender (male) | 1.544 (0.773, 3.084) | 0.219 |  |  |
| Hematemesis | 3.717 (1.834, 7.536) | **<0.001** | 4.682 (1.944, 11.273) | **<0.001** |
| Ascites | 0.707 (0.351, 1.424) | 0.331 |  |  |
| Corrected BMI<21.9 | 5.675 (2.673, 12.049) | **<0.001** | 6.196 (2.635, 14.568) | **<0.001** |
| Platelet >54.5 | 2.273 (1.055, 4.896) | **0.036** | 2.441 (0.907, 6.567) | 0.077 |
| ALT >2ULN | 1.627 (0.144, 18.369) | 0.694 |  |  |
| AST >2ULN | 1.366 (0.313, 5.953) | 0.678 |  |  |
| ALB < 35 g/L | 0.875 (0.440, 1.741) | 0.704 | 0.676 (0.201, 2.274) | 0.527 |
| Creatinine > 1ULN | 0.000 (0.000, -) | 1.000 |  |  |
| Urea nitrogen > 1 ULN | 0.912 (0.397, 2.095) | 0.828 |  |  |
| APTT > 1 ULN | 0.648 (0.324, 1.294) | 0.219 |  |  |
| Fibrinogen > 2.26 | 2.469 (1.236, 4.933) | **0.010** | 4.053 (1.543, 10.642) | **0.005** |

Note: Odds ratios and corresponding 95% confidence intervals were estimated using binary logistic regression analysis. Age and Child–Pugh score were retained in the multivariable model a priori as clinically relevant covariates. Other candidate variables with P <0.05 in the univariable analysis were entered into a backward likelihood-ratio multivariable logistic regression model. The removal and re-entry criteria were P >0.05 and P <0.05, respectively. OR: Odds ratio; CI: confidence intervals; BMI: body mass index; ALT: Alanine aminotransferase; AST: Aspartate aminotransferase; ALB: Albumin; APTT: activated partial thromboplastin time; ULN: upper limit of normal. Bold values indicate *p*< 0.05.

**Supplementary figure 1**

**Supplementary Figure 1** Relationships between skeletal muscle index (cm^2^/m^2^) and age.

**Supplementary figure 2**

**Supplementary Figure 2** The assessment of the nomogram for predicting myopenia in validation cohort. (A) The ROC of the nomogram in the development cohort. (B) Calibration curve of the development cohort. (C). Decision curve analysis for development cohort. AUC: Area under the curve; BMI: Body mass index; ROC: Receiver operating characteristic.
